# Clinical and surgical outcome in patients with cervical spondylodiscitis—a single-center retrospective case series of 24 patients

**DOI:** 10.3389/fsurg.2024.1292977

**Published:** 2024-06-03

**Authors:** S. Motov, B. Stemmer, P. Krauss, M. N. Bonk, C. Wolfert, K. Steininger, E. Shiban, B. Sommer

**Affiliations:** ^1^ Klinik für Neurochirurgie, Kantonsspital St. Gallen, St. Gallen, Switzerland; ^2^ Klinik für Neurochirurgie, Universitaetsklinik Augsburg, Augsburg, Germany

**Keywords:** epidural abscess, spinal, discitis osteomyelitis, sepsis, classification, spondylodesis

## Abstract

**Objective:**

Cervical spondylodiscitis is a rare pathology, with an incidence of 0.5–2.5 per 100,000 population, posing significant potential risks. This type of infection can lead to neurological impairment in up to 29% of patients. Radical surgical debridement of the infected segment, fusion, and an intravenous antibiotic regimen remains the gold standard in most spine centers. This study aimed to analyze the surgical outcome in a tertiary spine center based on disease severity.

**Methods:**

In this study, we retrospectively included all patients diagnosed with cervical spondylodiscitis and treated at the University Hospital Augsburg between January 2017 and May 2022. We collected and analyzed baseline parameters on clinical presentation with symptoms, laboratory parameters, radiological appearance, and surgical parameters such as type of approach and implant, as well as neurological and radiological outcomes. Descriptive statistics were performed using SPSS, and relevant correlations were examined using the *t*-test for independent samples and the chi-square test.

**Results:**

Twenty-four patients (9%) with cervical spondylodiscitis were identified. Twenty-two (92%) surgically treated patients were subdivided into the complicated discitis group (*n* = 14, 64%) and the uncomplicated discitis group (*n* = 8, 36%). Seventeen patients (71%) presented with sepsis on admission, 17 patients (71%) were diagnosed with epidural abscess on primary imaging, and 5 patients (21%) had more than one discitis lesion at a distant spinal segment. The presence of epidural abscess was significantly associated with systemic sepsis (OR = 6.2; *p* = 0.03) and myelopathy symptoms (OR = 14.4; *p* = 0.00). The most frequently detected specimen was a multisensitive *Staphylococcus aureus* (10 patients, 42%). Six patients (25%) died after a median of 20 days despite antibiogram-accurate therapy, five of whom were diagnosed with a complicated type of discitis. The follow-up data of 15 patients (63%) revealed permanent neurological damage in 9 patients (38%). Notably, the surgical approach was a significant factor for revision surgery (*p* = 0.008), as three out of five (60%) ventrodorsal cases with complicated discitis were revised.

**Conclusion:**

Cervical spondylodiscitis represents a severe infectious disease that is often associated with permanent neurological damage or a fatal outcome, despite adequate surgical and antibiotic treatments. Complicated types of discitis may require a more challenging surgical and clinical course.

## Introduction

Cervical spondylodiscitis is a rare pathology, with an incidence of 0.5–2.5 per 100,000 population (1). It appears to be particularly dangerous as it has a higher potential for permanent neurological damage compared to thoracolumbar spondylodiscitis (2). The mortality rate in individual case series varied between 5% and 10% (3, 4). Moreover, the occurrence of associated epidural abscesses, vertebral osteomyelitis, and meningitis might complicate the further recovery process and remain challenging factors (5). The development of complex deformities is another feared complication in the context of extensive infection in the mobile spine. Radical surgical treatment of spinal infection with fusion of the affected segments and subsequent antibiotic therapy remains the gold standard in most spine centers (4, 6). Even though it appears counterintuitive, previous studies demonstrated the safety and usability of implants in the context of spinal infections (7, 8). However, the type of implant (vertebral body replacement or intersomatic cage), implant material (PEEK or titanium), the extent of surgery (anterior approach, posterior approach, or combined surgery), and duration of antibiotic treatment are still center-specific variables and there is no consensus on the exact treatment algorithm (3, 4, 9–11). Nevertheless, some authors demonstrated that an anterior approach only might be a sufficient treatment and surgical therapy maintains a favorable outcome with improvement or return to normal neurological function in at least 66% of all patients (12). Since there were several changes in implants and a paradigm shift toward a more aggressive surgical treatment in recent years in our clinics, we explored the surgical outcome of primary cervical spondylodiscitis based on disease severity in a tertiary spine center.

## Methods

The study protocol was approved by the local ethics committee at Ludwig Maximilian University (No. 22-0585) in accordance with the Declaration of Helsinki. A retrospective analysis was performed on all patients who were treated either surgically or conservatively for primary cervical spondylodiscitis in the neurosurgical department of the University Hospital Augsburg from January 2017 to June 2022. The patients were included based on radiological evidence (cervical MRI w/o contrast enhancement) for cervical spondylodiscitis, clinical symptoms (myelopathy, radiculopathy, and/or neck pain), and laboratory results on admission (elevated CRP marker/leukocytosis—CRP > 5 mg/dl, leukocytes > 10 × 10^3^/µl). The exclusion criteria were prior cervical surgeries in the cervical spine or soft tissue, e.g., neck dissection and thyroid surgery. The rationale for surgery was predominantly based on neurological deficits (myelopathy, radiculopathy), intractable neck or arm pain, and elevated or rising inflammation markers under antibiotic treatment. The surgical approach and implants were based on the surgeons' preferences. The clinical parameters included patient demographics, surgery duration, and postoperative complications based on Clavien–Dindo classification (CDC), duration of hospital stay, intensive care treatment, duration and type of antibiotic treatment, and permanent neurological deficits. The preoperative (MRI/CT), immediate postoperative (x-ray/CT), and follow-up imaging (x-ray/CT/MRI) were analyzed with regard to implant failure, e.g., cage subsidence or migration. Additionally, the segmental sagittal correction was calculated based on the measurement of the local sagittal angle on x-ray imaging. The mean local sagittal angle was measured between the upper endplate and the lower endplate of the adjacent vertebrae. Subsidence was defined as implant migration or implant collapse >2 mm in the endplate on either CT or x-ray imaging.

**Figure 1:**
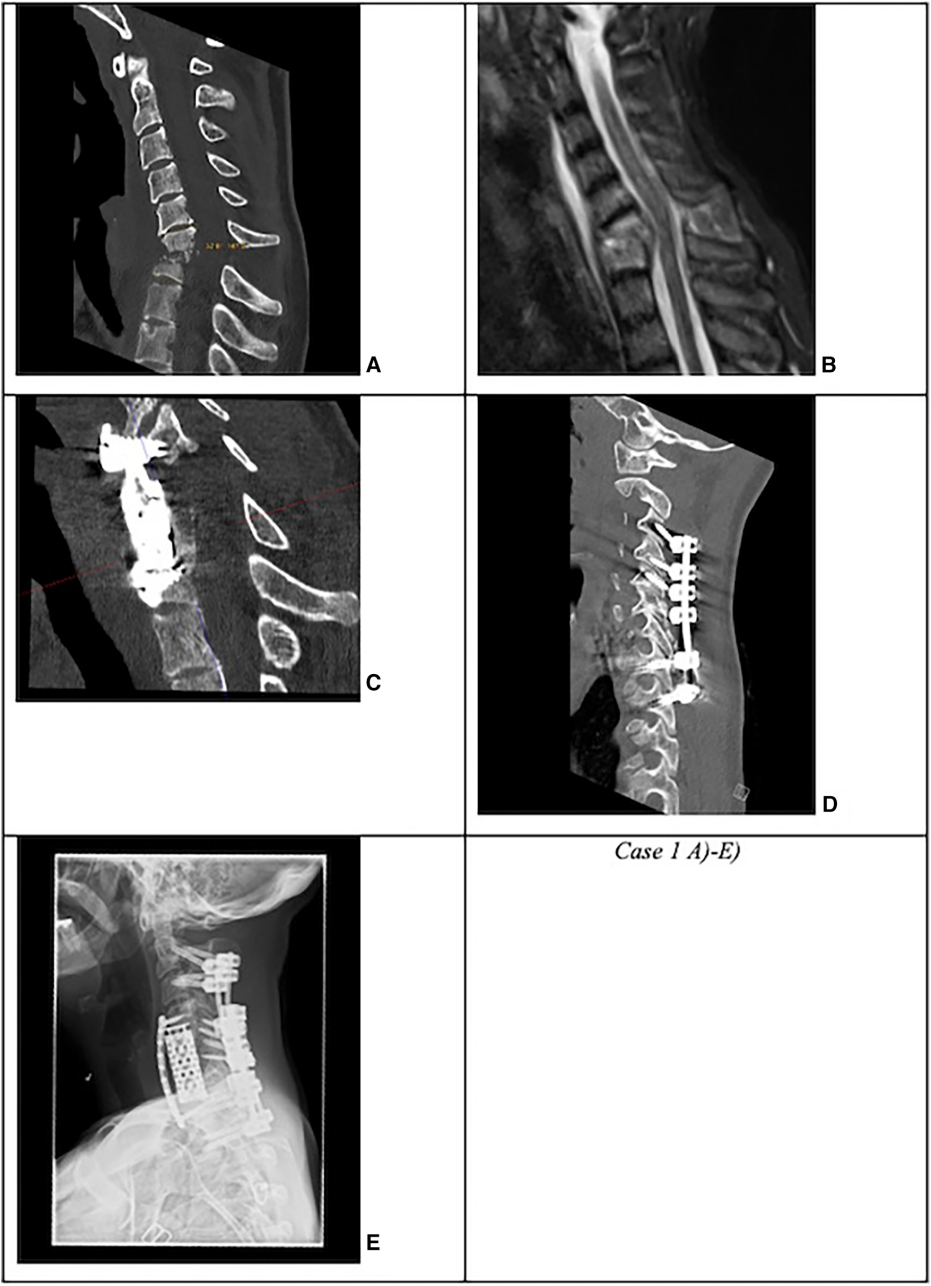
Case 1: Complicated cervical spondylodiscitis in C6/7 with long-segment (three levels) posterior epidural abscess, kyphotic sagittal deformity with sagittal angle of 33°, and progressive osteomyelitis in a drug abuser. (**A**) CT and (**B**) MRI with C6/7 spondylodiscitis with 33° kyphotic deformity. (**C**) First revision surgery due to cage subsidence with dorsal spondylodesis extension to C4 and anterior plating. (**D**) Secondary lateral mass screw loosening in C3 and C4. (**E**) Second revision surgery with pedicle screw placement in C2 and C3.

**Figure 2:**
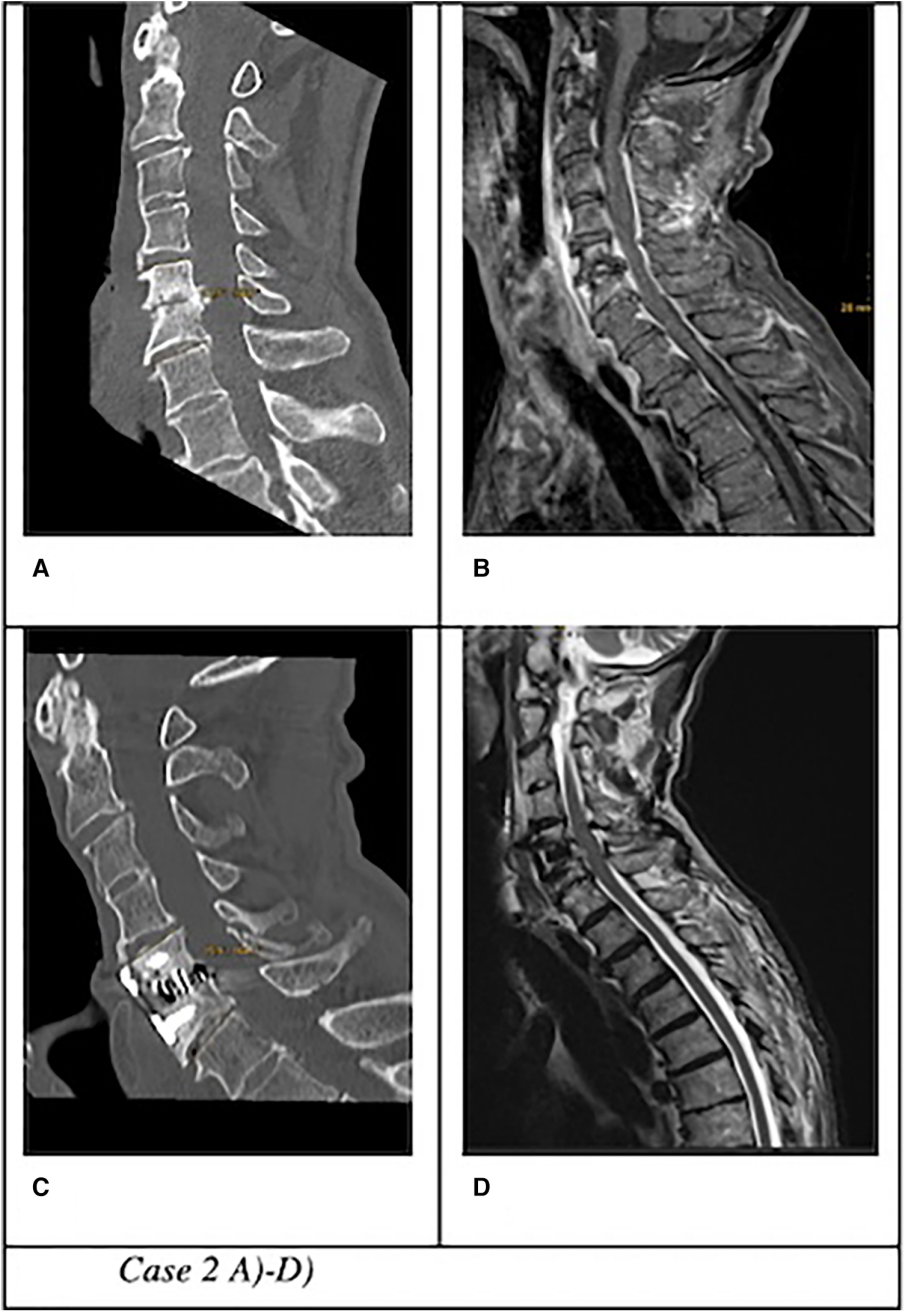
Case 2: Uncomplicated cervical spondylodiscitis at C5/6 in a patient with Parkinson's disease, local sagittal angle of 16°, no epidural abscess. (**A**) CT and (**B**) MRI of an 80-year-old patient with swallowing problems, fever, and elevated CRP markers treated with ACDF with anterior plating. (**C**) CT and (**D**) MRI on follow-up 3 months after surgery with bony fusion and no signs of infection.

Based on the number of affected levels (>1 level vs. 1 level), presence of kyphotic deformity (segmental sagittal angle of >12° vs. ≤12° between the upper endplate of the adjacent upper vertebra and the lower endplate of the lower adjacent vertebra), or presence of multi-segment (>1 vs. 1 level) epidural abscess, we subclassified the surgically treated patients into complicated and non-complicated discitis groups. The patients were included in one of the two groups if at least ≥2 criteria were confirmed (Figure 1, *Case 1* and Figure 2, *Case 2*).

Descriptive statistics were performed using SPSS Statistics (version 28, IBM Corp, Armonk, NY, USA), and relevant significant associations were obtained with a *t*-test for independent samples and a chi-square test. Data in text and graphs are expressed as median or mean and standard deviation (SD) with confidence interval (CI). A *p*-value of ≤0.05 was considered statistically significant.

## Results

### Baseline parameters

**Figure 3:**
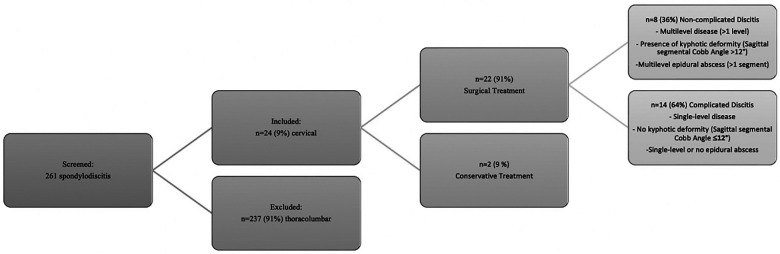
Screening and treatment flowchart.

**Figure 4:**
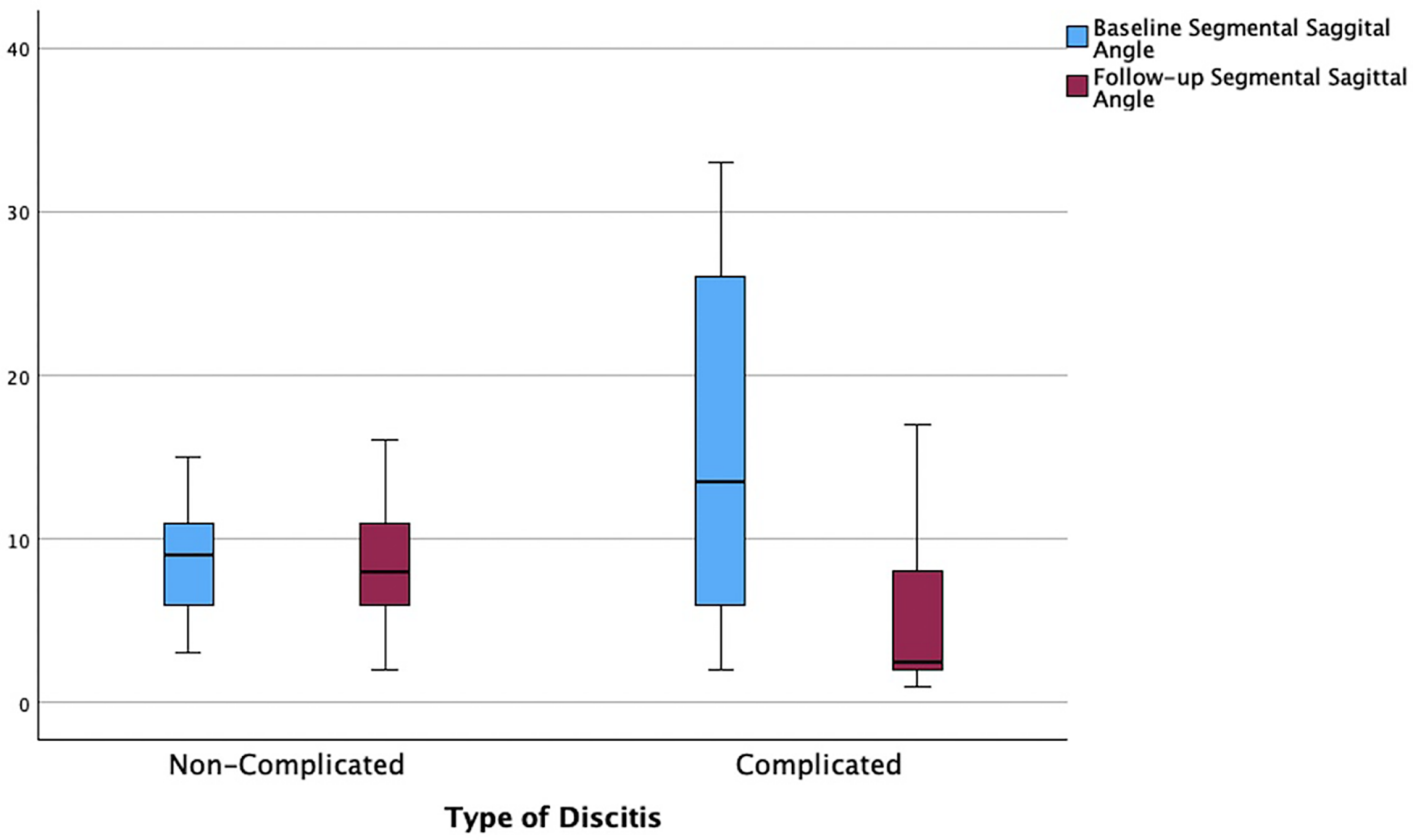
Correction of segmental sagittal angle from baseline to follow-up based on the type of discitis (*p* = 0.211).

We identified a total of 261 patients with spondylodiscitis in this period. Twenty-four patients (9%) with primary cervical spondylodiscitis were further included in the final analysis (Figure 3). Twenty-two patients (92%) were treated surgically, and two patients (8%) were treated conservatively. Eight (36%) of the surgically treated patients were diagnosed with a non-complicated type of discitis, and 14 patients (64%) were identified with a complicated type of discitis. In total, 17 patients (71%) had an epidural abscess on primary MRI imaging, with more patients (*n* = 11, 50%) being in the complicated discitis group (*p* = 0.17). The mean age was 66 ± 15 years, and there were predominantly male patients (*n* = 17, 71%). Nineteen patients (79%) presented with intractable neck pain, 15 patients (63%) with radiculopathy, and 13 patients (54%) with signs of myelopathy on admission. More patients in the complicated discitis group presented with either radiculopathy (*p* = 0.43) or myelopathy (*p* = 0.076), however without statistical significance (Table 1). Seventy-one percent of all patients were stratified with an American Society of Anesthesiologists (ASA) risk classification score of >3 corresponding to a severe systemic disease and 29% with an ASA score of 4 corresponding to a potentially life-threatening condition (Table 2). The median Charlson Comorbidity Index (CCI) averaged 5, indicating a 1-year mortality rate of 85% (13). Sixteen patients (67%) were either smokers or drug or alcohol abusers. The initial mean segmental sagittal angle was 12° (5; 17) with a mean local sagittal angle of 9° (1;12) in the non-complicated discitis group and 14° (1; 35) in the complicated discitis group (*p* = 0.211) (Figure 4).

**Table 1:** Surgical and neurological outcomes.

	Complicated discitis (*n* = 14)	Uncomplicated discitis (*n* = 8)	Total (*n* = 22)
Subsidence rate	4 (25%)	3 (19%)	7 (29%)
			(*p* = 0.38)
Revision rate	4 (18%)	0	4 (18%)
			(*p* = 0.095)
Mortality	5 (23%)	0	5 (23%)
			(*p* = 0.05)
Neurological Deficits	9 (41%)	4 (18%)	13 (59%)
			(*p* = 0.43)
Radiculopathy			
Myelopathy	9 (41%)	2 (9%)	11 (50%)
			(*p* = 0.076)
Epidural Abscess	11 (50%)	4 (18%)	15 (68%)
			(*p* = 0.17)

**Table 2:** Patient demographics.

Age (year)	66 ± 15
Gender	
Male	17 (71%)
Female	7 (29%)
BMI (kg/m^2^)	25 (23; 27)
Charlson Comorbidity Index (CCI)	5 (5; 6)
American Society of Anesthesiologists (ASA)	3 (3; 4)
Septic state	71% (*n* = 17)
Multifocal discitis (incl. other than C-spine)	21% (*n* = 5)
Number of segments	2 ± 1
Presence of epidural abscess	71% (*n* = 17)
Surgical times	
Anterior approach	170 min (62; 360)
Posterior approach	44 min
Combined approach	388 min (311; 490)
Clavien–Dindo Score (CDS)	2 ± 2
Inward stay	23 days (14; 31)
Intensive care unit stay	3 days (1; 8)
i.v. antibiotic treatment	20 days (13; 35)
p.o. antibiotic treatment	42 days (28; 42)

### Microbiology

**Figure 5:**
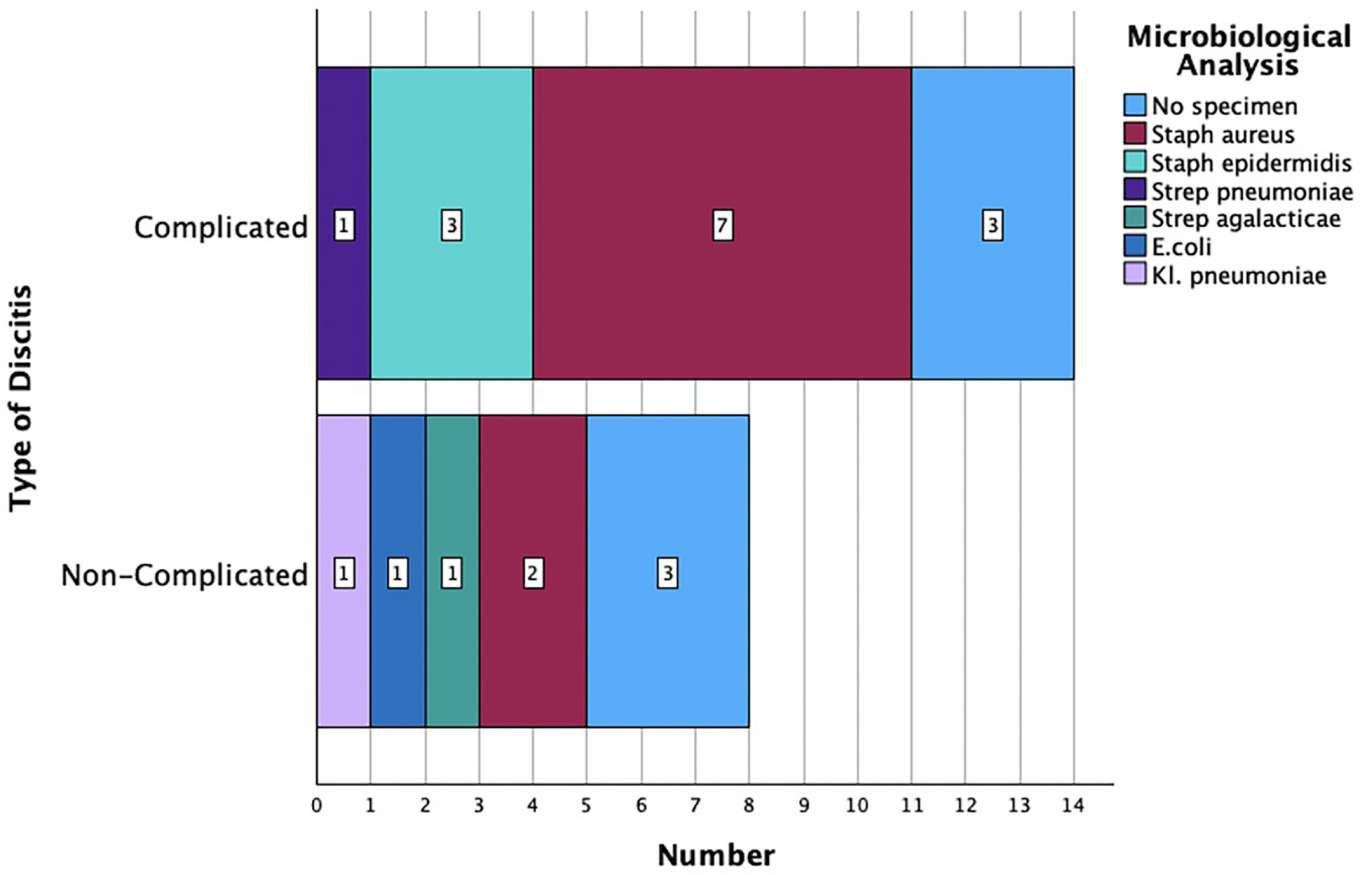
Microbiological analysis based on the type of discitis (*p* = 0.185).

Seventeen (71%) patients suffered from sepsis upon admission, which was defined based on two or more SIRS criteria (fever, >38.0 °C; hypothermia, <36.0 °C; tachycardia, >90 beats/min; tachypnea, >20 breaths/min; leukocytosis defined as a white blood cell count of >11 × 109 per L). All intraoperative probes of the surgically treated patients (*n* = 22; 92%) were sent for microbiological analysis, and those of 16 patients (67%) were also referred to histopathological assessment. A bacterial specimen was successfully identified in 18 of 22 patients (82%) in the microbiological analysis. Signs of chronic infection were detected in all histologically acquired specimens. The most common bacteria was a multisensitive *Staphylococcus aureus* (10 patients, 42%). There was no significant difference between the microbiological spectrum in the complicated and the non-complicated groups (*p* = 0.185) (Figure 5). The detailed microbiological analysis is presented in Table 3. Antibiotics were applied intravenously for a median duration of 20 days (CI 3; 53) and orally for a median of 42 days (CI 21; 63).

**Table 3:** Distribution of microbiological results.

	Number of patients/percentage (%)
*Staphylococcus aureus*	10 (42)
*Staphylococcus epidermidis*	3 (13)
*Streptococcus pneumoniae*	1 (4)
*Streptococcus agalactiae*	2 (8)
*Escherichia coli*	1 (4)
*Klebsiella pneumoniae*	1 (4)
No specimen	6 (25)
Total	24 (100)

### Surgical outcome

**Figure 6:**
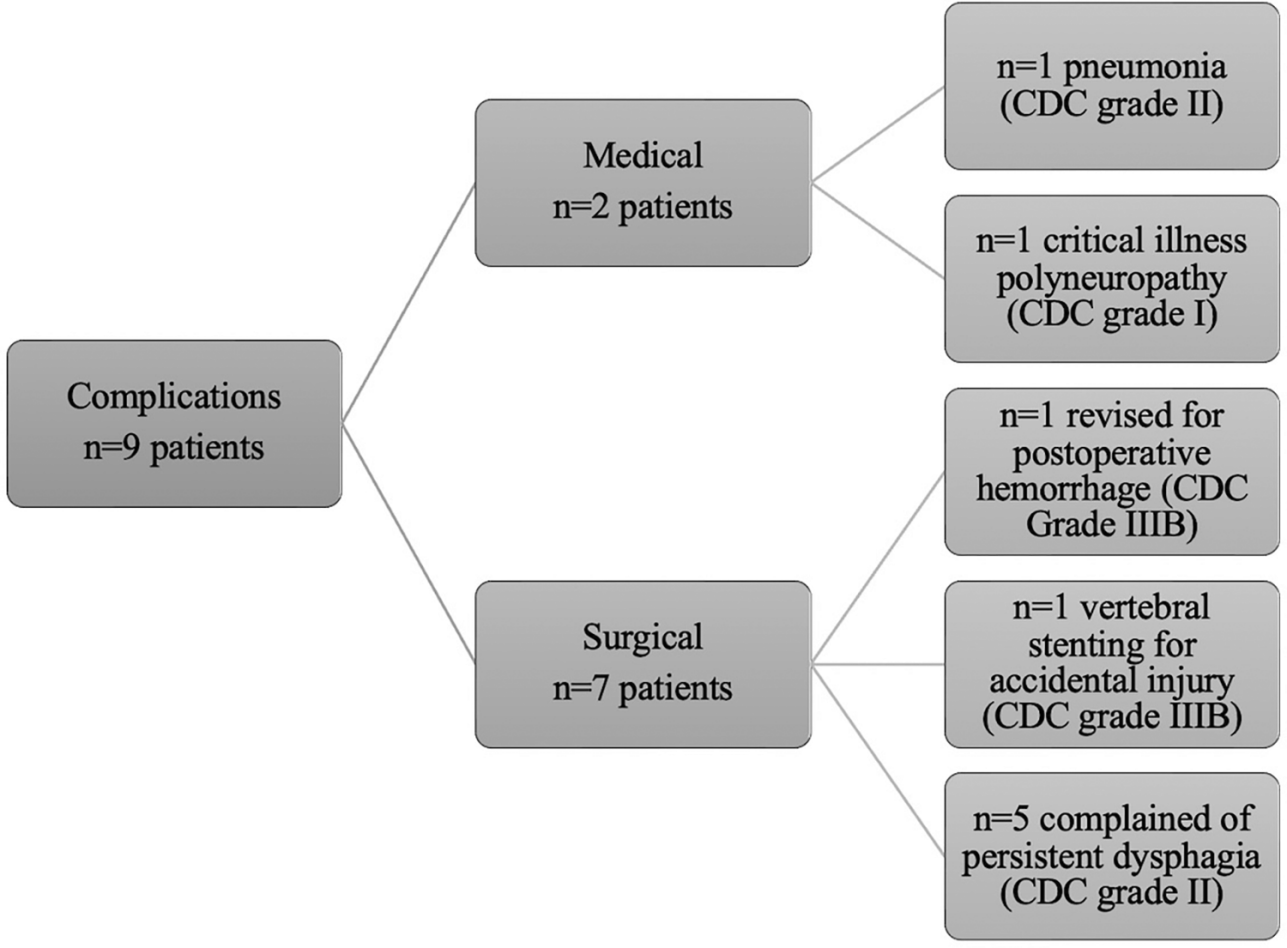
Type of complications during and after surgery based on CDC.

**Figure 7:**
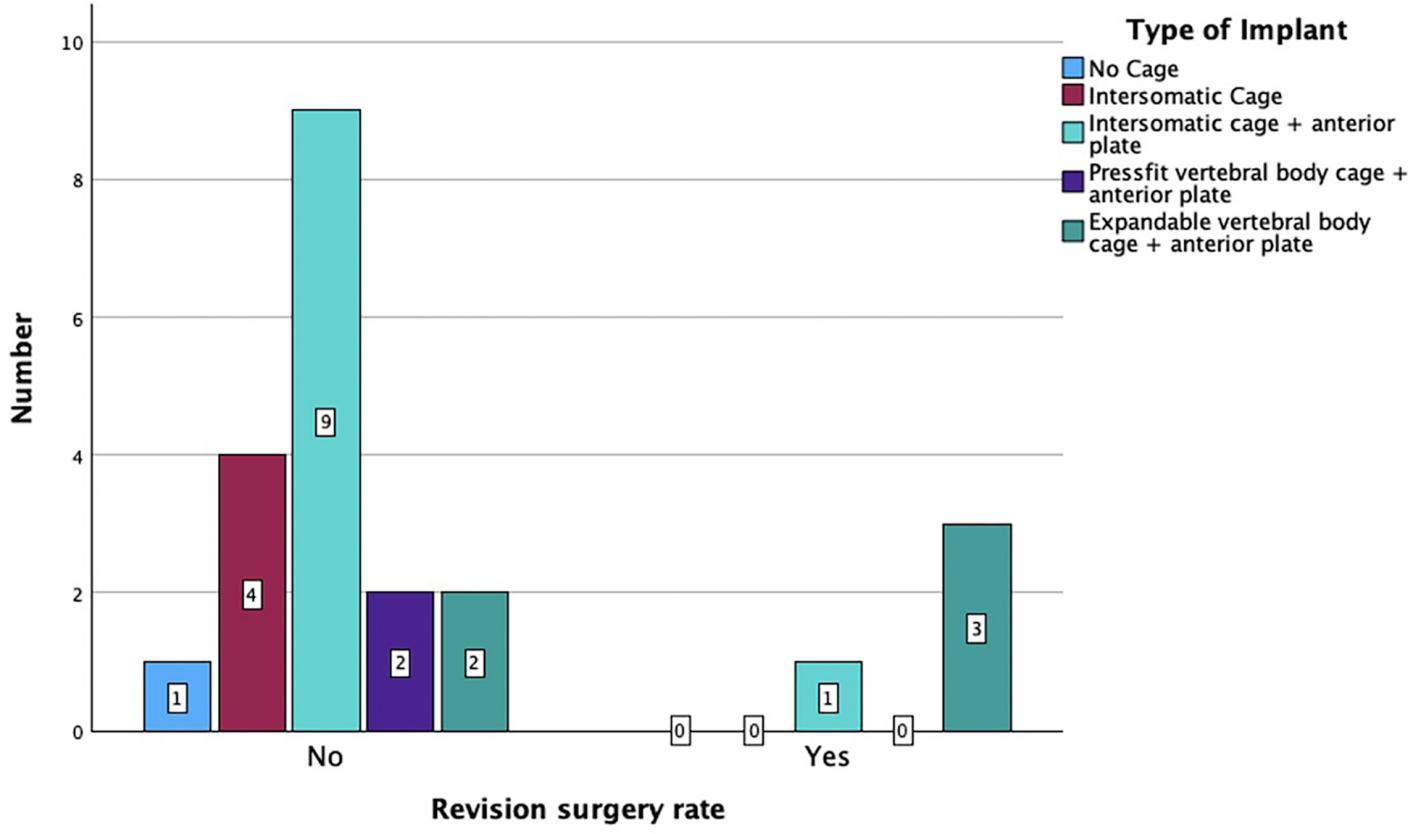
Revision surgery rate based on the type of implant (*p* = 0.92).

**Figure 8:**
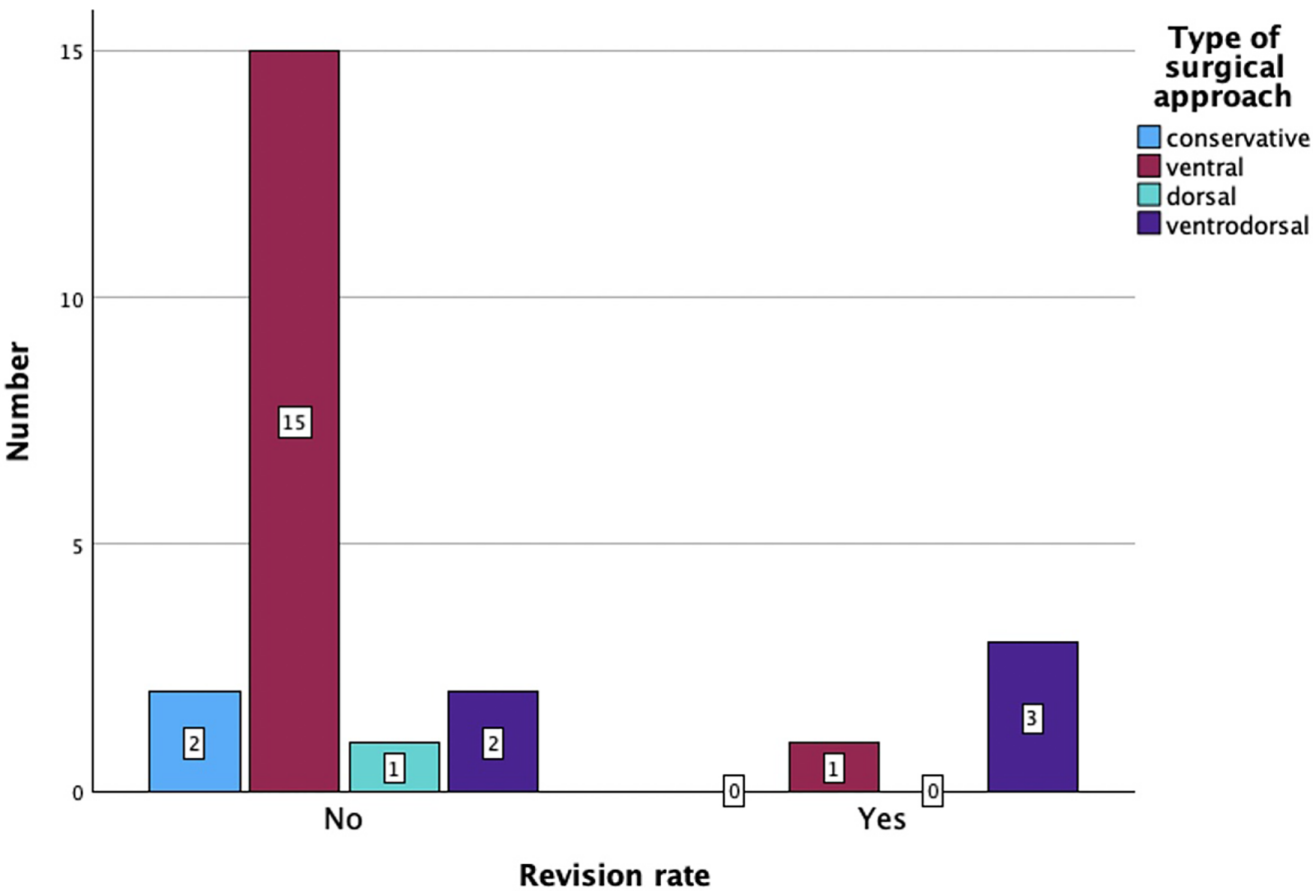
Revision surgery rate based on the surgical approach (*p* = 0.04).

Sixteen patients (67%) underwent anterior surgery, five patients (21%) underwent combined anterior and posterior fusion, and only one patient (4%) underwent posterior surgery. The exact type of implant, implant material, and approach are listed in Table 4. The type of complications that occurred during and after surgery as well as during the postoperative course of treatment are described in Figure 6. All patients with non-complicated discitis were treated with an anterior approach (*p* = 0.095) with either a stand-alone intersomatic cage (*n* = 4; 50%) or an intersomatic cage with anterior plate (*n* = 4; 50%) (*p* = 0.02). Surgical times differed based on the selected approach as the longest surgical times were experienced with combined approaches (*p* < 0.001) (Table 2). Radiological follow-up data were obtained from 15 surgically treated patients (63%). Eight patients received a CT, six patients received an anteroposterior and lateral x-ray, and one patient received an MRI after a median of 4.5 months (CI 1; 13). The mean local sagittal angle has been more successfully corrected in the complicated discitis group (5°) than in the non-complicated group (8°), however without significance (*p* = 0.255). Seven patients (29%) presented with cage subsidence on follow-up CT imaging with three patients (14%) from the non-complicated group and four patients (17%) from the complicated discitis group (*p* = 0.377). All four patients (17%) with complicated discitis required revision surgery (CDC grade IIIb) (*p* = 0.095) (Table 5). Three of them were revised for cage subsidence with migration after a median of 4 days. One of them was revised a second time despite a combined approach with posterior instrumentation due to a screw loosening in the upper instrumented vertebrae 171 days after initial surgery (Figure 1, *Case 1*). The microbiological results of the sonification probes in this case were negative. There was no difference based on the implant material regarding subsidence (*p* = 0.205) or revision rate (*p* = 0.916). Furthermore, the type of osteosynthesis (anterior plating, intersomatic cage, expandable vertebral body cage, combined anterior plating, or posterior screw-rod spondylodesis) was not significantly associated with subsidence (*p* = 0.13) or revision surgery (*p* = 0.20) in our cohort (Figure 7). The surgical approach was a significant factor for revision surgery (*p* = 0.008) as three out of five (60%) combined ventrodorsal cases were revised (Figure 8).

**Table 4:** Implant selection (all patients in the combined approach group were treated with posterior instrumentation with M.U.S.T Mini, Medacta)**.**

Approach	Implant name	Company	Implant material (cage)	Type of implant	Number of patients	Number of patients with anterior plate	Implant name (cage)	Company (cage)
Anterior	Cervicus Invadur	PINA	PEEK	Intersomatic cage	6	5	SHARK	PINA
	Sourire Cage	Arca Medica			2	1		
	Mecta-C Cage	Medacta			1	1	Mecta-C	Medacta
	Hygro-C	Nexon Medical	Titanium		5	3	1 × Skyline	DePuy Synthes
							2 × Tryptik	SpineArt
	Capri Cage	Tsunami Medical			3	2	1 × SHARK	PINA
							1 × Tryptik	SpineArt
Combined	ADD	Ulrich		Vertebral body replacement	2	2	Tryptik	SpineArt
	Cervilift	PINA			2	2	1 × SHARK	PINA
							1 × Tryptik	SpineArt
Posterior	M.U.S.T Mini	Medacta	Titanium	Screw-rod instrumentation	1	0		
Total					22	16		

**Table 5:** Hardware revision characteristics.

Indication for revision	Δ Segmental angle correction^a^	Construct length	Cage level	Number of instrumented segments	Age (year)	BMI (kg/m^2^)	ASA	CCI	Drug abuse (incl. nicotine/alcohol)
Screw loosening	4°	C3-Th2	C3-C6	6	76	24	3	6	0
Cage subsidence	28°	C2-Th2	C3-Th1	7	41	23	3	3	1
	15°	C4-Th2	C5–7	5	69	25	3	3	1
	15°	C4–C7	C4–7	3	60	29	2	5	1

^a^ Difference between angles measured from the upper endplate to the lower endplate of the affected vertebrae before and after surgery.

### Clinical follow-up data

Clinical follow-up data were obtained from 15 patients (14 surgically/1 conservatively treated; 63%) who had a complete recovery from the infection with a median follow-up of 8 months (CI 1; 13). One of the conservatively treated patients refused to be operated upon and died despite being timely treated in an intensive care unit with broad-spectrum antibiotics. The other conservatively treated patient was ineligible for surgery due to poor clinical condition and survived with conservative treatment for 8 months, but long-term follow-up data were missing. Six patients (25%) died after a median follow-up of 20 days (CI 4; 926) past initial diagnosis despite calculated antibiotic therapy due to sepsis with multiorgan failure. The type of discitis was a nearly significant factor (*p* = 0.054) for a death-related outcome since all surgically treated patients who later died were classified with complicated discitis (*n* = 5; 21%). Permanent neurological damage such as radiculopathy (*n* = 4; 17%), myelopathy (*n* = 5; 21%), or complete sensorimotor cross-section (*n* = 2; 8%) was noted in nine patients (38%) on follow-up. Two patients (8%) had a late relapse of spondylodiscitis affecting a lumbar spinal segment after a median of 741 days (24 months). Due to differences in the standard of care in the initial 2 years of this study, only 10 patients (42%) received a holospinal MRI on admission or during their hospital stay, which revealed a second distant discitis lesion (thoracolumbar location) in 5 patients (21%). The presence of an epidural abscess was significantly associated with sepsis (OR = 6.2; *p* = 0.03) and myelopathy symptoms (OR = 14.4; *p* = 0.00). Sepsis was also significantly associated with the occurrence of discitis in other segments (*p* = 0.02), higher CCI (*p* = 0.03) and Clavien–Dindo scores (*p* = 0.01), and a longer ICU stay (5 days vs. 7 days, *p* = 0.04). Age was significantly associated with a death-related outcome (*p* = 0.03). There was a trend for death-related outcomes based on the extent of infection without statistical significance (*p* = 0.09). Higher ASA (*p* = 0.04) and CDC (*p* = 0.01) scores were also significant risk factors, and higher CCI (*p* = 0.08) was a trending factor for death-related outcomes in our cohort.

## Discussion

### Clinical outcomes based on discitis severity assessment

We retrospectively reviewed the clinical and radiological parameters of patients treated for primary cervical spondylodiscitis in a 5-year period at a single tertiary spine center. Cervical spondylodiscitis has been previously described as a rare condition (3, 5, 14, 15) diagnosed in only 9% of our patients. However, improvement in imaging diagnostics and increasing population age might lead to a rising prevalence of this spine infection in general (16). The clinical characteristics of the patient cohort in our study corresponded to the results of prior metanalyses and retrospective case series (4, 12, 16). Most patients in this study were male (71%); were severely ill (71% with an ASA score of >3 and median CCI score of 5), consumed nicotine, drugs, or alcohol (67%); and presented with an epidural abscess (71%) and septic status (71%) on admission. The presence of an epidural abscess complicated the course of treatment and was significantly associated with sepsis and myelopathy symptoms, which have been previously described (5, 12, 14, 16). Even though one-third of the patients in our study had a non-complicated single-segment disease, more than one-third developed persistent and debilitating neurological deficits, and a quarter of all patients even died during further course of treatment, which appears to be a higher number than previously reported. Age, higher ASA and CDC scores, and the grading in a complicated type of discitis were related to a poorer clinical outcome in our study. The most common reason for a fatal outcome was a multiorgan failure resulting from sepsis. We also observed a prolonged hospitalization phase, which might further aggravate preexisting medical conditions eventually leading to more complications, especially in the elderly population. Prior studies favored a conservative treatment in the absence of neurological deficits, sufficient pain control, and non-existing epidural abscess or significant deformity (12, 17). Criteria such as the presence of a septic condition, long-segment epidural abscess, and severe kyphotic deformity with local sagittal angle >12° were important factors in our cohort in determining the further course of treatment. The combination of these characteristics also led to a higher rate of revision surgery or a death-related outcome. Preexisting scores such as the Brighton spondylodiscitis score (BSDS) estimated parameters such as cervical localization and complete sensorimotor deficits with lower figures (18), which appears to be inadequate in our study population. Additionally, the BSDS could not be internationally validated (19). We suggest that a more elaborate grading system similar to the MSI-20 score, which includes clinical, laboratory, radiological, and frailty parameters, should be applied and further refined in cervical spondylodiscitis cases (20). The development of hyperkyphotic deformities such as in Figure 1, *Case 1*, may additionally cause permanent neurological deficits, chronic neck pain, and loss of the horizontal visual axis, which also emphasizes the need for a lower threshold for surgical correction and internal fixation to prevent deformity progression. A more comprehensive discitis subtype classification might eventually help in assessing the risks and benefits of surgery and in establishing the surgical approach.

### Radiological and histopathological findings

Although only 10 patients (42%) in our series were examined with a whole spinal MRI, half of them were diagnosed with a further infectious focus in a distant spinal segment. As a result, there is a high probability that several patients were underdiagnosed in the past and suffered from spondylodiscitis in further segments, which were not treated surgically. Previous studies recommend a complete spine MRI since the rate of multilocal spondylodiscitis might range between 6.8% and 12.7% (21). Even though cervical spondylodiscitis healed, two patients developed a late relapse in distant segments in our series. This might be related to preexisting medical conditions with a higher mortality rate based on CCI in our cohort. The predominant microbiological spectrum in our study included multisensitive *Staphylococcus aureus* (42%) and *Staphylococcus epidermidis* (13%), which corresponds to the clinical findings in previous literature (4). The microbiological pathogen was similar in both groups of complicated and uncomplicated discitis. Although several patients had major medical comorbidities and there were some alcohol and drug abusers, we could not identify any patients with multidrug-resistant bacteria in our cohort.

### Surgical approaches and hardware complications

The patients in this study were treated with various types of implants since surgical concepts and implants have changed several times in our clinics. However, there was no correlation between these parameters and the rate of hardware failures in the short-term follow-up. There was no significant difference between the subsidence rate of PEEK and titanium cages, which has also been described previously (11). In general, the rate of hardware complications including the cage subsidence rate of 29% and the surgical revision rate of 17% appears to be high in this particular patient cohort, which corresponds to the findings from previous real-world data (3, 4, 9). Despite this, only four patients (18%) in the complicated discitis group required revision surgery in our clinics due to implant subsidence with migration. Some authors propose a 360° fusion if there is severe bony destruction, huge surgical defects with an incredibly destabilized spine, and poor bone quality or if the patient's compliance with an external orthosis is low in this particular patient group (5). Still, our results confirm that in most cases especially with non-complicated discitis, intersomatic vertebral cages with or without plating regardless of implant material might be a sufficient surgical option (Figure 2, *Case 2*). In cases with multilevel infection and kyphotic deformities, a higher extent of the spondylodesis and even a combination with posterior instrumentation might be mandatory. Nevertheless, combined approaches correlated with a higher chance of revision surgery in our cohort. All our combined surgery cases were treated in a staged manner with a mean of 5 days (CI 2–10) between both surgeries. It remains debatable whether the anterior and posterior approaches should be performed in a staged manner or simultaneously (3, 10). In our exemplary Figure 1, *Case 1*, the patient developed cage subsidence of a combined expandable vertebral body cage with anterior screw system (ADD+, Ulrich) 9 days after initial surgery and complained of swallowing problems. It remains questionable if posterior instrumentation during the initial surgery might have prevented this complication. Previous studies demonstrated that combined ventrodorsal approaches in infectious cases might be associated with a higher rate of hardware complications than in degenerative or trauma cases (4), which corresponds to our observations.

## Limitations

A specific limitation of this study was the retrospective character and the heterogeneous diagnostic and treatment data as well as the limited number of patients. Only 10 patients (42%) received a holospinal MRI, which is a standard diagnostic imaging tool in most spine centers nowadays (22). Exact surgical treatment and the type of implants varied on surgeons' preferences and local clinical standards. Another limitation was the incomplete follow-up data with only 15 patients receiving follow-up imaging and clinical consultation after discharge. Additionally, the short clinical and radiological follow-up period of 8 and 4.5 months, respectively, makes it difficult to explore the rate of non-union and total hardware failures. The small amount of conservatively treated patients and the overall low number of patients further restricts the validity of our conclusions. Indeed, it seems difficult to vindicate a higher number of conservatively treated patients taking into consideration the results of potential neurological impairment and disease progression under conservative treatment, especially in the complicated type of discitis. A multicenter study with preestablished treatment modalities and higher patient numbers might overcome these limitations.

## Conclusion

In conclusion, cervical spondylodiscitis is a severe infectious disease often associated with septic state and epidural abscesses. It frequently occurs in multimorbid elderly patients and, despite adequate surgical and antibiotic treatment, might often lead to permanent neurological damage and a fatal outcome. Anterior fusion with plating was a sufficient surgical approach in most non-complicated discitis cases in our study. In complicated multilevel discitis with long-segment abscesses or kyphotic deformities, a combined ventrodorsal approach might be mandatory. A higher rate of hardware complications with the need for revision surgery and close monitoring should be taken into consideration in these cases.

## Data Availability

The raw data supporting the conclusions of this article will be made available by the authors, without undue reservation.
